# Malignant pericardial effusion: sclerotherapy or local chemotherapy?

**DOI:** 10.1038/sj.bjc.6605108

**Published:** 2009-06-09

**Authors:** C Lestuzzi, C Lafaras, A Bearz, R Gralec, E Viel, A Buonadonna, T Bischiniotis

**Affiliations:** 1 Department of Cardiology, Centro di Riferimento Oncologico, IRCCS, National Cancer Institute, Aviano (PN), Italy; 2 Department of Cardiology, Theagenion Cancer Hospital, Thessaloniki, Greece; 3 Department of Oncology, Centro di Riferimento Oncologico, National Cancer Institute, Aviano (PN), Italy; 4 Cardio-Pulmonary Intensive Care, National TB and Lung Disease Research Institute, Warsaw, Poland

Sir,

We have read with great interest the article of Kunitoh *et al*, 2009 published recently on this Journal, about the use of intrapericardial Bleomycin in malignant pericardial effusion (MPE) with lung cancer. We appreciate the effort of trying a prospective, randomised study in this field, but we do not agree with the rationale and the method of the study.

First of all, the title is misleading, as the Authors also include cytologically negative effusions among the ‘malignant effusions’, without any mention about other means to confirm their malignant nature. At least 30% of pericardial effusions in lung cancer patients are not due to metastases, and these cases should be examined separately (Wang *et al*, 2000; Porte *et al*, 1999). Actually, when the Authors did so in the subgroup analysis, the difference between the two groups of treatment is evident (even without a statistically significant difference). A title as: ‘Pericardial effusion in lung cancer patients’ should be more appropriate.

Second, in the introduction, the Authors assume that the therapy of malignant pericardial effusion is, by definition, pericardial sclerosis, and include the use of various chemotherapeutic agents such as ‘sclerosing’. Actually, Bleomycin has both antineoplastic and sclerosing (with a mechanism analogue to the tetracyclines) properties, whereas platinum, thiotepa and vinblastine are actually ‘pure’ antineoplastic drugs. In fact, the goal of the use of these agents is not to simply prevent mechanically the accumulation of the pericardial fluid, but try to cure the pericardial metastases. And this leads to the third, and more important point.

Malignant pericardial effusion is a metastatic localisation, and, in our opinion, the goal of treatment should be to try to cure it with antineoplastic agents rather than simply prevent their secondary effects. Echocardiographic evaluation in many lung cancer patients detects discrete pericardial implantations or infiltrations, and in certain patients, diffuses neoplastic deposits. Thus, local chemotherapy has the rationale to control not only the pericardial fluid re-accumulation but the neoplastic process as well. The injection of a chemotherapeutic agent in a limited space, such as the pericardium, with heart's movement allowing the diffusion of the agent to the whole surface, and the slow re-absorption through the lymphatic vessels (the main diffusion way of metastases to the pericardium in lung and other cancers), has several advantages: high intrapericardial concentration of chemotherapy (CT) for several days, low blood concentration (and few systemic side effects) and beneficial effects on the lymphatic system obstruction (Figoli *et al*, 1987; Reynen *et al*, 2004; Tomkowski *et al*, 2004). On the contrary, the exaggerated sclerosing process after intrapericardial instillation is the main problem of sclerosing agents, such as bleomycin. The risk of sclerosing therapy is in fact not only the evolution to constrictive pericarditis (as already reminded by the Authors), but also to effusive-constrictive pericarditis (where even small amount of fluid leads to tamponade because of the reduced compliance of the thickened pericardium), with consequently problems in attempting a second drainage in case of haemodynamic impairment if the effusion is loculated. In the report by Kunitoh *et al*, among 79 patients, two cases of constrictive pericarditis and two deaths of massive bleeding ‘during attempt of re-drainage …possibly due to crack formation in the ventricular wall upon dissection of the adherent pericardium’.

We and others have reported the low complication rate and the effectiveness of local chemotherapy (Lestuzzi *et al*, 2000; Maisch *et al*, 2002; Martinoni *et al*, 2004; Tomkowski *et al*, 2004; Bischiniotis *et al*, 2005). In our personal experience of 139 cases of MPE due to various neoplasms (88 lung cancer) and treated with local chemotherapy (platinum in most cases, given either as single 50 mg in 50 ml of saline in single bolus or as 10 mg in 20 ml of saline over 3–5 running days), we had no major complications except for: one case of renal failure (treated with fluid and furosemide), one severe chest pain with electrocardiogram abnormalities (but no troponine increase), one atrial fibrillation requiring DC shock for cardioversion and two late constrictive pericarditis after local CT with platinum. A minority of patients complained of pain or nausea. We had no problems in any of the eight patients who underwent a second pericardiocentesis. In addition, in one of our Institutions (Theagenion Cancer Hospital) 15 lung cancer patients underwent serial cytological examination of the pericardial fluid that showed a remarkable neoplastic burden reduction after the third dose of cisplatin. This acute response of local chemotherapy may predict its long-term favourable effects.

About the outcome, our experience is encouraging. In all tumours, the mean effusion-free period of the patients treated with local chemotherapy was 372 days, median 223; at 1, 2, 6 and 12 months, 58, 52, 33 and 16%, respectively, were completely effusion-free. In the subgroup of lung cancer MPE, the mean effusion-free period was 271 days (median 215) and the percentage of completely effusion-free at 1, 2, 6 and 12 months was 65, 57, 35 and 18%, respectively; also including the patients who had persistent mild effusion (without haemodynamic impairment). The success rate in this subgroup raises to 86, 70, 40 and 19%, respectively. Our results are then slightly better, on the long-term period, when compared with those of Kunitoh (who reports an effusion-free survival of 65, 46, 24 and 10% at 1,2,6 and 12 months, respectively), with fewer complications.

In conclusion, we feel that sclerosing therapy (with any agent) should not be considered as the first choice treatment for MPE anymore. The use of local CT agent is safer, more rationale and more effective. Coping with lung cancer, platinum is the first-choice drug for systemic therapy (and, logically, for local therapy as well) (D'Addario and Felip, 2008).
